# Elasmobranch qPCR reference genes: a case study of hypoxia preconditioned epaulette sharks

**DOI:** 10.1186/1471-2199-11-27

**Published:** 2010-04-23

**Authors:** Kalle T Rytkönen, Gillian MC Renshaw, Kevin J Ashton, Grant Williams-Pritchard, Erica H Leder, Mikko Nikinmaa

**Affiliations:** 1 Division of Genetics and Physiology, Department of Biology, University of Turku, FI-20014 Turku, Finland; 2 Centre of Excellence in Evolutionary Genetics and Physiology, Department of Biology, University of Turku, Turku, Finland; 3 Hypoxia and Ischemia Research Unit, School of Physiotherapy and Exercise Science, Griffith University, Queensland, Australia; 4 Biomedical Science, Bond University, Queensland, Australia; 5 Heart Foundation Research Centre, Griffith University, Queensland, Australia

## Abstract

**Background:**

Elasmobranch fishes are an ancient group of vertebrates which have high potential as model species for research into evolutionary physiology and genomics. However, no comparative studies have established suitable reference genes for quantitative PCR (qPCR) in elasmobranchs for any physiological conditions. Oxygen availability has been a major force shaping the physiological evolution of vertebrates, especially fishes. Here we examined the suitability of 9 reference candidates from various functional categories after a single hypoxic insult or after hypoxia preconditioning in epaulette shark (*Hemiscyllium ocellatum*).

**Results:**

Epaulette sharks were caught and exposed to hypoxia. Tissues were collected from 10 controls, 10 individuals with single hypoxic insult and 10 individuals with hypoxia preconditioning (8 hypoxic insults, 12 hours apart). We produced sequence information for reference gene candidates and monitored mRNA expression levels in four tissues: cerebellum, heart, gill and eye. The stability of the genes was examined with analysis of variance, geNorm and NormFinder. The best ranking genes in our study were *eukaryotic translation elongation factor 1 beta *(*eef1b*), *ubiquitin *(*ubq*) and *polymerase (RNA) II (DNA directed) polypeptide F *(*polr2f*). The performance of the *ribosomal protein L6 *(*rpl6*) was tissue-dependent. Notably, in one tissue the analysis of variance indicated statistically significant differences between treatments for genes that were ranked as the most stable candidates by reference gene software.

**Conclusions:**

Our results indicate that *eef1b *and *ubq *are generally the most suitable reference genes for the conditions and tissues in the present epaulette shark studies. These genes could also be potential reference gene candidates for other physiological studies examining stress in elasmobranchs. The results emphasise the importance of inter-group variation in reference gene evaluation.

## Background

Whilst the majority of all the fish species belong to teleost fishes, there is increasing evolutionary, conservational and economic interest in the elasmobranch fishes [1,2]. Elasmobranchs are at the base of the gnathostome lineage as the oldest extant group of jawed vertebrates - they diverged from the lineage leading to tetrapods and teleosts approximately 450 million years ago (MYA) [3]. From a genomic perspective cartilaginous fishes, including elasmobranchs, have advantages as model species compared to teleosts [4]. This is because teleost specific genome duplications are absent from cartilaginous fishes and the tetrapod gene homologs are comparable in a more direct manner. A preliminary genome draft is available for cartilaginous chimaera, the elephant shark [4], but chimaeras and elasmobranchs are estimated to have diverged 375 MYA [5]. At present there are no genome sequences available for elasmobranchs and the most convenient way to study mRNA expression is the quantitative PCR (qPCR) technology [6], where carefully selected target genes can be monitored.

In qPCR the amount of mRNA can be monitored by absolute or relative quantification. Absolute quantification can be achieved, for example, by using an external RNA control [7] or by using cRNA standards for the target gene [8]. The relative quantification is based on the expression ratio of mRNAs of a target gene and an internal reference gene (or "housekeeper" gene) [6,9]. Both means of quantification have their advantages. We chose to use the relative quantification with endogenous reference genes, because this also corrects experimental variation resulting from quality of the samples, pipetting errors and reverse transcription efficiency. The choice of a suitable reference gene or genes is crucial for the accuracy of the analysis. The reference gene should have constant mRNA expression levels in all the conditions or treatments under study. For each new experimental design the suitability of various potential reference genes needs to be tested and validated [10].

Oxygen has been a major force shaping the evolution of vertebrates, especially fishes, which have a diverse array of physiological adaptations evolved in response to reduced oxygen availability [11-13]. As hypoxia is a very strong stress factor, genes belonging to a number of functional categories are expected to be regulated in the course of hypoxic insult. This applies also to the potential qPCR reference genes and, based on literature; it is challenging to shortlist recommendable reference gene candidates for hypoxia studies. Hypoxia responses have been studied in teleosts, e.g. cyprinids [14], often using *actin *as a reference gene. However, recent studies on fishes suggest that *actin *is generally not an optimal reference gene [10,15-17]. Furthermore, *eukaryotic translation elongation factor *has been commonly used, but it has been reported to be up-regulated in prolonged hypoxia in zebrafish gills [14]. For genes encoding ribosomal proteins, which are commonly used as reference gene candidates, microarray studies have revealed a hypoxic mRNA down-regulation in zebrafish gills [14] and in *Xiphophorus maculatus *tissues [18], but conversely there was an hypoxic mRNA up-regulation in zebrafish heart [19]. Thus, it is clear that the performance of the reference gene candidates is tissue-dependent.

The epaulette shark (*Hemiscyllium ocellatum*) is often exposed to intermittent hypoxic periods in its natural environment during nocturnal low tides [20]. Since laboratory preconditioned epaulette sharks have demonstrated improved tolerance to hypoxia as a result of respiratory and metabolic adjustments [21], we administered a laboratory-based hypoxic preconditioning protocol to prime protective responses in these animals. This protocol consisted of 8 successive exposures to severe sub-lethal hypoxia (5% of full air saturation) every 12 hours. We then studied four tissues: cerebellum, heart, gill and eye. These tissues were selected because they all have specific responses which enable the animals to survive hypoxia and even anoxia. The gills can compensate for oxygen availability [22] and the heart can conserve energy during hypoxia [23]. If oxygen levels are depleted cerebellar shut down conserves brain energy charge [20] and also the retinal light response is temporarily suppressed [24].

For elasmobranchs, there are no previous reports comparing reference gene candidates or reported collections of reference gene sequences available for any physiological stress. Here we comprehensively tested 9 reference genes that were selected across various functional categories, taking into account the literature on hypoxia studies and reference gene comparisons in other aquatic vertebrates. To our knowledge, this was the first study to produce sequence information on reference gene candidates for an elasmobranch, the epaulette shark, and to identify suitable reference genes for different tissue types in hypoxia and hypoxia preconditioning studies. Stability of the genes was evaluated with available software for this purpose [25,26] in addition to basic analysis of variance and finally the results of the different statistical approaches were compared.

## Results

### Novel reference gene sequences for an elasmobranch species

A novel set of epaulette shark reference gene candidate sequences was collected for this study (Table 1). These candidates range across various functional categories and are not expected to be co-regulated. Partial sequences for *DNA J subfamily A2 *(*heat shock protein 40*) (*dnaja2*), *hypoxanthine phosphoribosyltransferase 1 *(*hprt*), *myosin phosphatase-rho interacting protein *(*mrip*) and *polymerase (RNA) II (DNA directed) polypeptide F *(*polr2f*) and *tubulin beta 2 *(*tubb2*) were obtained with universal primers based on cross-vertebrate alignment and *ubiquitin *(*ubq*) qPCR primer was successfully designed directly from the cross-vertebrate alignment. The GenBank accession numbers of the sequences obtained in this study are shown in Table 1. As the evolutionary distance from elasmobranchs to tetrapods and teleosts is considerable (we tested various universal primer combinations per gene and many were unsuccessful), we expect that these sequences will aid primer design for specific qPCR also in other elasmobranch species in addition to the epaulette shark used here. Primers for *cardiac alpha-actin *(*actc1*), *60S ribosomal protein L6 *(*rpl6*) and *eukaryotic translation elongation factor 1 beta *(*eef1b*), were designed from cDNA library clone sequences originating from cardiac muscle.

**Table 1:** Candidate qPCR reference genes tested in this study with the putative function of the proteins they encode and accession numbers of the produced epaulette shark gene sequences.

Symbol	Gene (homolog) name	Function	Genbank accession
*actc1*	*alpha-actin, cardiac muscle 1*	Structural protein	GU983379
*dnaja2*	*dnaJ subfamily A 2 (Hsp40)*	Chaperone	GQ152302
*eef1b*	*eukaryotic translation elongation factor 1 beta*	Protein synthesis	GU983380
*hprt*	*hypoxanthine phosphoribosyltransferase 1*	Purine nucleotide synthesis	GQ152303
*mrip*	*myosin phosphatase-Rho interacting protein*	Component of the myosin phosphatase complex	GQ152304
*polr2f*	*polymerase (RNA) II (DNA directed) polypeptide F*	Component of RNA polymerase II	GQ152305
*rpl6*	*60S ribosomal protein L6*	Component of the large 60S ribosomal subunit	GU983381
*tubb2*	*tubulin beta 2*	Structural protein	*
*ubq*	*ubiquitin*	Protein degradation	GQ152306

* qPCR primer designed directly from the *tubb2 *alignment of following vertebrate species: human ENST00000340384, chicken ENSGALT00000022476, frog ENSXETT00000047287, stickleback ENSGACT00000016810, zebrafish BC062827.1

### mRNA expression levels in cerebellum, heart, gills and eye

**Figure 1:**
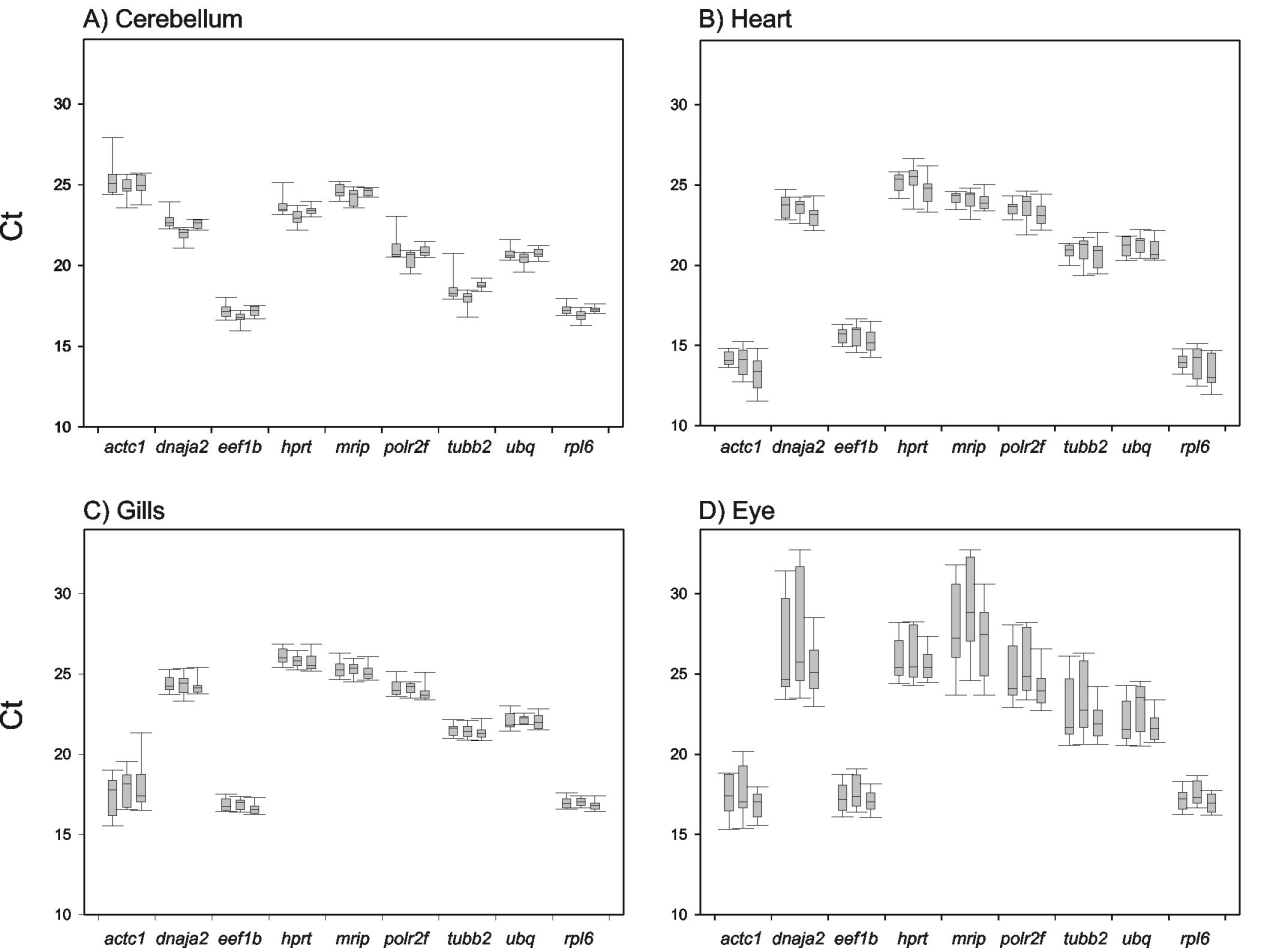
**The mRNA expression levels of the genes studied are represented by the raw Ct values**. A) Cerebellum B) Heart C) Gills D) Eye. For each gene the three experimental conditions are displayed from left to right in three box-plots: control, single hypoxic insult and hypoxia preconditioning (8 hypoxic insults, 12 hours apart). The box indicates the 25/75 percentiles and the line marks the median.

The reference gene candidates were studied in a sample series collected from cerebellum, heart, gills and eyes. In the final analysis each of the four tissue series included 10 individuals from each treatment (control, single hypoxic insult and hypoxia preconditioning), except that there were only 9 hearts and eyes from the control group and 9 eyes from the single hypoxic insult. The mRNA expression levels of the genes in different treatments can preliminary be illustrated with the raw Critical threshold (Ct) values (Figure 1). Ct value is inversely related to the abundance of the specific gene transcript and indicates the PCR cycle at which the fluorescence intensity equals the selected threshold fluorescence. The Ct values of the samples varied between 11.5 (heart, *actc1*) and 32.7 (eye, *dnaja*). The mRNA expression levels of individual genes were relatively similar in all four tissues for most of the genes. Highest expression difference was observed in *actc1 *between cerebellum (app. 25) and heart (app. 13). When the variation between samples among tissues within a treatment was compared, there was small variation in cerebellum, small or medium in heart and gills, and very large in eyes. It should be noted that the removal of the retina during eye sampling may have introduced some sampling-dependent variation which does not reflect natural biological variation. The highest intra-treatment individual variation was observed in *actc1 *and lowest in *rpl6*.

### Stability analysis and suitability of the reference gene candidates

After descriptive analysis of the Ct values the data were transformed to relative quantities with standard curve method. We first analysed among group variation with analysis of variance (ANOVA, parametric) for control group and the two treatments (Table 2). Generally, the reference gene candidates were stable among the conditions. In heart, gills and eye there were no significant differences with ANOVA (p < 0.01 or p < 0.05) (Table 2). In cerebellum four genes (*dnaja2, hprt, rpl6 *and *tubb2*) showed significant variation among the experimental groups (p < 0.01). For *dnaja2, hprt *and *rpl6*, when treatments were tested pair-wise with ANOVA, the mRNA expression after a single hypoxic insult was statistically different from that of control and of hypoxia-preconditioned animals. There were no significant differences in mRNA expression between hypoxia-preconditioned and control animals.

**Table 2:** Results of the mRNA expression stability analysis with NormFinder and GeNorm with ANOVA tests for differences among experimental groups.

Tissue/Rank	NormFinder (Stability value)		GeNorm (M value)	
Cerebellum				

1	dnaja2**	0.043	eef1b* rpl6**	0.25
2	hprt**	0.045	ubq*	0.30
3	ubq*	0.061	dnaja2**	0.32
4	*polr2f*	0.067	hprt**	0.34
5	rpl6**	0.068	*polr2f*	0.37
6	eef1b*	0.070	tubb2**	0.40
7	tubb2**	0.079	*mrip*	0.45
8	*mrip*	0.121	*actc1*	0.49
9	*actc1*	0.122		

Heart				

1	*eef1b*	0.058	*eef1b ubq*	0.18
2	*polr2f*	0.070	*hprt*	0.21
3	*mrip*	0.080	*polr2f*	0.23
4	*ubq*	0.086	*mrip*	0.26
5	*tubb2*	0.093	*tubb2*	0.28
6	*dnaja2*	0.109	*dnaja2*	0.31
7	*hprt*	0.114	*actc1*	0.38
8	*actc1*	0.175	*rpl6*	0.45
9	*rpl6*	0.183		

Gills				

1	*rpl6*	0.044	*eef1b rpl6*	0.14
2	*polr2f*	0.064	*polr2f*	0.19
3	*eef1b*	0.066	*dnaja2*	0.23
4	*tubb2*	0.073	*tubb2*	0.24
5	*ubq*	0.084	*hprt*	0.26
6	*dnaja2*	0.084	*ubq*	0.28
7	*mrip*	0.089	*mrip*	0.29
8	*hprt*	0.111	*actc1*	0.51
9	*actc1*	0.277		

Eye				

1	*ubq*	0.087	*eef1b rpl6*	0.21
2	*polr2f*	0.110	*hprt*	0.42
3	*tubb2*	0.117	*ubq*	0.48
4	*hprt*	0.193	*polr2f*	0.54
5	*actc1*	0.210	*actc1*	0.58
6	*eef1b*	0.227	*tubb2*	0.62
7	*rpl6*	0.253	*dnaja2*	0.77
8	*dnaja2*	0.297	*mrip*	0.99
9	*mrip*	0.341		

* ANOVA p < 0.05

** ANOVA p < 0.01

Next we analysed the data with two algorithms designed for reference gene analysis, NormFinder and geNorm [25,26]. The model-based method in NormFinder takes into account both the intra-group and inter-group variation (stability value). The geNorm input does not have the ability to separate experimental groups so its stability ranking reflects the total variation present in the data set. The results of these analyses are shown in ranked order (Table 2). In heart, gills and eye NormFinder and geNorm rankings did not contradict the ANOVA tests. However, in cerebellum the two top ranking candidates from the NormFinder analysis (*dnaja2, hprt*) and one top ranking candidate of geNorm (*rpl6*) were removed because of ANOVA (p < 0.01). After this, in cerebellum both methods ranked *ubq, eef1b *and *polr2f *as the best candidates. In heart the best results according to NormFinder were *eef1b, polr2f *and *mrip *whereas according to geNorm the best candidates were *eef1b, ubq *and *hprt*. In gills according to both methods *rpl6, eef1b *and *polr2f *were the top candidates and in eye NormFinder selected *ubq, polr2f *and *tubb2 *whereas geNorm selected *eef1b*, *rpl6 *and *hprt*. On the basis of all of the results from all tissues the consensus was that *ubq, eef1b *and *polr2f *were generally the best reference gene candidates in the conditions studied. In contrast, *actc1 *was the worst candidate.

NormFinder calculates a stability value estimate for the best combination of two reference genes. The best two gene combinations were in cerebellum (after removing the ANOVA hits) *eef1b-polr2f *(0.047), in heart *eef1b-mrip *(0.041), in gills *rpl6-tubb2 *(0.043) and in eye *ubq-polr2f *(0.063). geNorm has the function to estimate the usefulness of adding extra reference genes for optimal result in qPCR studies. This estimation is obtained by analysis of the changes in normalization factors when e.g. the third ranking gene is added to the set of the two best candidates. In our data sets the addition of a third gene did not make a considerable difference if the recommended [25] cut-off of 0.15 was used. The exception was the eye where the addition of a third gene could be useful (0.017).

The NormFinder and geNorm ranking contradicted ANOVA test only in cerebellum where the intra-group variation was the lowest. We carefully investigated ANOVA tests in cerebellar data sets in more detail. The model assumes that the error term should follow the assumptions for a univariate measurement process. After performing an analysis of variance, the model should be validated by analyzing the residuals. For *dnaja2, hprt *and *rpl6*, we inspected the normality of the residuals graphically and performed normality and homogeneity tests (Table 3). The values in Table 3 were obtained using the raw relative quantity values, as the logarithmic and square transformations did not change the significance levels dramatically. In the case of *dnaja2 *and *hprt *both the normality and the homogeneity assumptions are fulfilled and ANOVA is valid. In the case of *rpl6 *the homogeneity assumption is not fulfilled (Levene test, p < 0.05), but based on the significance values and graphs of the residuals we predict that ANOVA results are still robust. In cerebellum also *ubq *and *eef1b *are moderately differential among treatments (p < 0.05) (Table 2), and there was a general trend (Figure 1A) that most genes were slightly up-regulated in the single hypoxic insult.

**Table 3:** Normality (Shapiro-Wilk) test on the residuals, homogeneity of variances test and ANOVA for cerebellum DNAJA2, HPRT and RPL6 data sets.

Test		*dnaja2*	*hprt*	*rpl6*
Normality (Shapiro-Wilk)	W	0.94	0.94	0.99
	df	30	30	30
	p	0.092	0.107	0.987
Homogeneity of variances	F	2.06	1.90	4.58
(Levene)	df	2; 27	2; 27	2; 27
	p	0.147	0.169	**0.019**
ANOVA	F	13.9	6.5	6.2
	df	2; 27	2; 27	2; 27
	p	**0.0001**	**0.0051**	**0.0059**

Values with p < 0.05 are displayed in bold

## Discussion

Cartilaginous fishes including elasmobranchs are the oldest extant group of jawed vertebrates and have slower rate of evolution and simpler genomic structure than teleost fishes [4]. Currently various teleost fish genomes are available, but no full genome information exist for elasmobranchs. In the absence of extensive genomic information, quantitative PCR will remain a practical choice for mRNA expression studies for this species group, especially if a small number of target genes are to be studied.

Here we have studied 9 potential reference genes for an elasmobranch species in response to hypoxia. To our knowledge this is the first study that evaluates the suitability of potential reference genes for qPCR studies in elasmobranchs. We expect that the sequences deposited to GenBank will aid primer design for additional elasmobranch species in addition to the species described here. For example, both epaulette shark and nurse shark (*Ginglymostoma cirratum*), for which a BAC library is available [27], belong to Orectolobiformes (carpet sharks). Since hypoxia occurs commonly in the aquatic environment [11] the reference gene candidates presented in this study may be useful for other hypoxia experiments in elasmobranchs that live in benthic and tidal zones. However, reference genes must be always be evaluated case-specifically for every biological condition or treatment.

Overall, we did not observe considerable between-treatment differences in the reference gene candidates. Only in cerebellum mRNA expression of a few reference gene candidates was up-regulated specifically in response to a single hypoxic insult, but this did not occur in response to hypoxic preconditioning. This may indicate that the protective mechanisms in the brains of epaulette shark are rapidly modified or fine-tuned in response to repeated exposures of hypoxia, as has been earlier suggested [20].

The performances of the 9 reference gene candidates remained relatively similar in the conditions examined. *Actin (actc1) *was the worst of the tested reference genes in this study and together with the previous reports [10,15] this suggests that *actins *may generally be non-optimal reference genes. *Beta-actin *has been previously used as a reference gene in elasmobranchs [28]. Also the gene encoding for *tubulin*, which is a structural protein of cells, was not ranked among the most stable genes in our study. Our results emphasise that in some tissues ribosomal binding protein genes are not optimal reference genes for hypoxia studies. In this study *rpl6 *could be recommended for gills and eye, but was not optimal for cerebellum and heart. For example in response to a single hypoxic insult, the mRNA expression level of *rpl6 *increased in cerebellum. Similarly, in the single hypoxic insult the mRNA expression level of *dnaja2 *increased in cerebellum. *Dnaja2 *is a chaperone, and the usefulness of chaperones as reference genes appears to be highly case-specific. In bladder cancer data sets, *heat shock protein cognate B *(*hspcb*) clearly outperformed other candidates [26]. In epaulette shark, hsp70 protein levels increased in anoxia but not in hypoxia [29]. However, in other species, heat shock protein levels often increase in hypoxia [30,31].

The three overall best ranking candidates in this comparison were *eef1b*, *ubq *and *polr2f *and they are all from different functional categories, including both catabolic and anabolic processes. *Ubiquitin *is involved in protein degradation, whereas *eukaryotic translation elongation factor 1 beta *is involved in protein synthesis and *polymerase (RNA) II (DNA directed) polypeptide F *(*polr2f*) in RNA synthesis. *Polr2f *is not widely used as a reference gene, but may hold potential for this purpose [32]. *Eef1b *[33-35] and *ubq *[15,16] have been reported as suitable references genes in several physiological settings in different teleosts. However, for longer periods of hypoxia there have been observations of mRNA expression changes in specific tissues. In zebrafish gills elongation factors were up-regulated after 25 day hypoxic exposure [14] and in medaka, liver *ubiquitin *was down-regulated after 6 days of hypoxia [36]. We emphasize that in response to long-term hypoxic exposures *eef1b *and *ubq *could perform differently from the results presented here.

Reference or housekeeping genes often have very high transcription levels compared to the target genes of interest. If we put weight on the expectation that the transcription level of the reference gene should be close to the transcription level of the target genes, then *ubq *or *polr2f *are preferred over *eef1b *(in cerebellum, heart and gills the Ct approximately 20-24 or 19-25 versus 14-19). For this reason we decided not to include *18S *or *28S rRNA *genes in our study, as their transcription levels are very high compared to the mRNAs of the target genes [37].

The geNorm analysis suggests that including a third reference gene is not very useful and based on NormFinder stability values the use of two reference genes simultaneously instead of one adds more reliability to the analysis. Since reference genes are always run on the same qPCR plate with the target genes, the inclusion of a second reference gene will also introduce a greater workload depending on the platform used.

NormFinder and geNorm performed very similarly in our data even though their underlying algorithms are very different [15,25,26]. This has been observed in various cases [15,35,38]. Previously, NormFinder has been recommended over other methods, like geNorm or Bestkeeper [39], because it takes account both the intra-group and the inter-group variation, whereas the latter methods do not have this ability [15]. In our cerebellum data set, it was striking that the analysis of variance indicated statistically significant differences between treatments for genes that were ranked as the most stable candidates by the reference gene software. In the case of geNorm, this can be explained by the absence of inter-group variation function, but for NormFinder analysis, this requires a more careful consideration. Our observation is not caused because of great intra-group individual variation due to the ecological sampling or laboratory procedures, because it was noticed only in cerebellum, and of the four studied tissues cerebellum had the lowest degree of intra-group individual variation. We carefully validated the ANOVA results and could conclude that at least in the cases of *dnaja *and *hprt *the NormFinder ranking did not perfectly reflect the apparently real inter-group variation in the data. In the cerebellum most genes, even in functionally different categories, were up-regulated in the single hypoxic insult in our data set. This may have influenced NormFinder ranking as it may conform to the mRNA expression pattern that the whole data set shares together [26]. In addition, NormFinder by default uses the logarithmic transformation of the raw relative quantities, whereas in ANOVA analysis we by default used the raw relative quantities if they did not violate normality assumptions.

In summary, the above mentioned observations indicate that it is always advisable to include basic statistical tests in the analysis of reference genes for qPCR. We do not suggest that the two specific reference gene analysis methods used here are unreliable, rather that in some data sets they may not perform optimally. Again, since geNorm does not have the ability to perform inter-group analysis, this limitation should be considered carefully. A detailed consideration of NormFinder statistical procedure is out of the scope of our study. However, as the data presented for the cerebellum suggests, it is possible that in some cases the NormFinder stability values may not allocate enough weight to the inter-group variation.

## Conclusions

Elasmobranch fishes are an ancient group of vertebrates and have high potential as model species for research into evolutionary physiology and genomics. Our results indicate that *eef1b, ubq *and *polr2f *are the most suitable reference genes candidates for qPCR studies of hypoxia responses in a hypoxia-tolerant elasmobranch, the epaulette shark. These genes could also be potential reference gene candidates for other physiological studies in elasmobranchs. In contrast, *actc1 *performed poorly and the performance of *rpl6 *varied among tissues. The results emphasise the importance of careful analysis of inter-group variation in reference gene evaluation.

## Methods

### Fish collection, hypoxia exposures and tissue collection

Epaulette sharks (*Hemiscyllium ocellatum*) (mean length 57 ± 4.9 cm) were collected from the reef platform surrounding Heron Island Research Station, (23°27 'S. 151°55 'E). Collection permits (G25214.1 and G04/12777.1) were obtained from the Great Barrier Reef Protection Authority. The sharks were held in a 1000 l flow-through holding pool at the ambient reef conditions of 25 ± 2 °C and 36‰ salinity, for 24 to 48 hours prior to the start of the experiments during which time they were not fed. Sharks were pair matched for sex, weight and length before being randomly assigned to either a single episode of hypoxia (n = 10), hypoxia preconditioning (8 hypoxic insults, 12 hours apart) or normoxic conditions as a control (n = 10). The hypoxic-preconditioning protocol used to elicit the shark's neuroprotective responses to hypoxia has been previously described [21,40,41]. Briefly, this consisted of two hours of hypoxia at 0.34 mg O_2_/l (5% of air saturation) using nitrogen gas to displace dissolved O_2_. This hypoxic exposure was repeated every twelve hours for four consecutive days, except for the group receiving a single episode of hypoxia. Immediately after the last hypoxic exposure, sharks were euthanased by anaesthesia (7.5 mls of 5% benzocaine in 1 litre of fresh seawater at 0.34 mg O_2_/l for hypoxia treated animals or at normoxia for control animals). The brains were rapidly removed and dissected to isolate the different part of the brain including cerebellum and eye, followed by the removal of gills, heart. All four tissues were frozen immediately in liquid nitrogen and then stored at -80 °C. Prior to RNA extraction the retina was removed from the eye cup and placed in Trizol while still frozen.

### RNA extraction and cDNA synthesis

Tissues were homogenized and RNA was extracted using TRI reagent (Sigma, Saint Louis, MI, USA) according to the manufacturer's instructions. RNA was treated with Turbo DNAse (Ambion, Austin, TX, USA). The integrity of the total RNA was assessed by agarose gel electrophoresis and quantified (A_260_/A_280_) spectrophotometrically (NanoDrop ND-1000). Total RNA (1,6 μg) was reverse transcribed with Super script III reverse transcriptase (Invitrogen, Carlsbad, CA USA) in the presence of RNAse inhibitor (RNAseOUT, Invitrogen) with a cocktail of oligoT (2 μM) and random primers (30 ng). The cDNA was stored in -20°C until the qPCR, for which it was diluted 40-fold for heart and cerebellum and 20-fold for gills and eyes.

### Reference gene sequences for epaulette shark

Part of the gene sequences used for qPCR primer design were *de novo *sequenced in epaulette shark with universal primers. Universal primers (Table 4) based on alignments of tetrapod (human, mouse, chicken, frog) and teleost (zebrafish, medaka, stickleback, fugu) sequences were designed using netprimer http://www.premierbiosoft.com/netprimer to obtain a primary sequence fragment for reference genes (Table 4). A "touch- down" protocol with 20 cycles of "touch-down" [denaturation at 94°C for 30 s, annealing for 30 s with temperature decreasing 0.5°C each cycle starting at 63°C and finishing at 53°C, extension at 72°C for 1 min] and 20 cycles of denaturation at 94°C for 30 s, annealing 30 s at 53°C, and extension at 72°C for 1 min was employed. PCR products were transferred and run on agarose gel, and bands of specific sizes were excised and extracted with a Montage DNA Gel Extraction Kit (Millipore, Bedford, MA, USA). The fragments were either sequenced directly or, when necessary, cloned with pGEM-T Easy Vector System I (Promega, Madison, WI, USA) and sequenced after colony PCR. Sequencing was done using the ABI PRISM™ BigDye Terminator v 3.1 Cycle Sequencing Kit (Applied Biosystems, Foster City, CA, USA). The identities of the sequences were verified in BLAST and with alignments by hand.

**Table 4:** Primers used in this study.

Gene	Universal primers Forward/Reverse	qPCR primers Forward/Reverse	amplicon (bp)	E
*actc1*	-	CACCTTCCAGCAGATGTGGAT TACTGGGCGGGACTGAGATT	101	0.93
*dnaja2*	YRTGCGBRTYATGATHAGACA GACMSAVCCTGGYTCRATDACTTT	AAGAAGGTGGTGAAAGAAGTGAAGA CAAAGTGCCTCAACAAGACCA	217	0.71
*eef1b*	-	GCTGTGGATGATGACGACGA GCACCCCAAAGTAAGCCATC	247	0.90
*hprt*	GCTGAYCTVYTDGAYTACATCAA GGGCRTADCCYACMACRAAYTTGTC	TCAATCCCAATGACGGTAGACTT AGTCTGCATTGTTTTACCCGTATC	161	0.83
*mrip*	GRGTRGAGAGYGGYTACTT CARTCRAAKGTYTTBGAGCG	ACAGTACCGCAGGGCAAAGT CCAATGCTTCTTCCACTGACC	126	0.92
*polr2f*	ATGTCNGAYAACGARGAYAAYTT YTCRTCMMMNCCCCAGTC	TGATGGAGATGACTTTGACGATG ACACGGGCTCGCTCATACTT	170	0.84
*rpl6*	-	CACCAGGAGGGCGAGATCT TGGTCATCCTTCCGCTGTTC	71	0.96
*tubb2*	*	CACCTTCATTGGCAACAGCAC GGCCTCTGTGAACTCCATCTC	98	0.91
*ubq*	CYGGSAAGACCATCACCCT GGTSGACTCYTTCTGGATGTTGTA	CTGGCAAACAGCTGGAAGA ATCTGCATGCCACCTCTCA	99	1.01

For part of the genes universal primers were used to acquire novel Epaulette shark sequences. The amplicon length and PCR efficiency (E) of qPCR primers is displayed.

* qPCR primer designed directly from the *tubb2 *alignment, see table 1

### Quantitative PCR

Based on the partial sequences of the reference gene candidates 3-6 primer combinations were designed for each gene (Primer3) [42] and tested. The length of some partial sequences restricted the primer design, so we allowed the amplicon length to vary between 70-250 bp to acquire optimal primer pairs. Also, since special attention to avoid contamination of genomic DNA was displayed in the phase separation step of phenol-chloroform extraction and the usage of rigorous DNAse treatment, the criterion for exon-exon boundaries in the primer design was relaxed when necessary. The specificity and size of the amplicons obtained with primer pairs was preliminary checked on an agarose gel and with qPCR dissociation curves. Also qPCR efficiency was taken into account when choosing the best pair for each gene (Table 4). Identities of the selected amplicons were verified by sequencing (BigDye Terminator v 1.1 Cycle Sequencing Kit).

Quantitative PCRs were run on 7900 HT Fast Real-Time PCR System (Applied Biosystems) with Maxima SYBR Green qPCR Master Mix (2 ×) (Fermentas, St.Leon-Rot, Germany) using 2-step protocol [initial denaturation at 95°C for 15 min, 40 cycles of denaturation at 95°C for 15 s and annealing/extension at 60°C for 1 min] with dissociation curve analysis and all primers at [75 nM]. The experimental sample series (3 × 10 individuals) was run in divided blocks [(3, 3, 4) (3, 4, 3) (4, 3, 3)] to avoid any spatial or temporal bias resulting from pipetting and time lags in qPCR runs. Samples were run in triplicates, with no-template controls/minus reverse transcriptase controls, and standard curves embedded in the experimental block design. For PCR efficiency calculations standard curves were constructed from five point dilution series (1/10 to 1/910) from pooled cDNA. In standard curves r^2 ^> 0.97, in a very few cases of r^2 ^> 0.94 the clear outliers replicates were removed from the final standard curve. The Ct values of the non template controls were generally not detectable. All experimental samples were inside the linear range of the standard curve.

### Data analysis

The primary analysis of the qPCR reactions including the conversion of Ct values to relative quantities using standard curves was done with ABI 7900HT Version 2.3 Sequence Detection Systems (Applied Biosystems). Relative quantity values were examined by analysis of variance (ANOVA) among the control and treatment groups, and then with pairwise testing. This was followed with NormFinder [26] and geNorm [25] analysis. NormFinder determines the stability of the genes taking account of both intra- and inter-group (treatment) variations. GeNorm determines the stability without input options to take account the groups (treatment) of the individuals in the study. The ANOVA analysis was supplemented with graphical analysis of the residuals, normality tests and homogeneity tests implemented in SPSS 11 (SPSS Inc.).

## Authors' contributions

KTR wrote the manuscript, analyzed the data, carried out the laboratory work, participated in the design of the study and participated in the animal collection and experiments. GMCR designed and carried out the hypoxia experiments and animal dissections, participated in the supervision and editing the manuscript. KA and GP provided some of the sequence and primer information and support in the laboratory. EHL participated in the design and supervision of the laboratory work, data analysis and editing of the manuscript. MN planned the overall research program of which this study is a part of, and participated in the design and finalizing of the manuscript. All authors read and approved the final manuscript.
